# Increased Frequency and Compromised Function of T Regulatory Cells in Systemic Sclerosis (SSc) Is Related to a Diminished CD69 and TGFβ Expression

**DOI:** 10.1371/journal.pone.0005981

**Published:** 2009-06-22

**Authors:** Timothy R. D. J. Radstake, Lenny van Bon, Jasper Broen, Mark Wenink, Kim Santegoets, Yanhui Deng, Anila Hussaini, Robert Simms, William W. Cruikshank, Robert Lafyatis

**Affiliations:** 1 Department of Rheumatology, Radboud University Nijmegen Medical Center, Nijmegen, The Netherlands; 2 The Arthritis Center, Boston University School of Medicine, Boston, Massachusetts, United States of America; 3 BUMC Flow Cytometry Core Facility, Boston University School of Medicine, Boston, Massachusetts, United States of America; 4 The Pulmonary Center, Boston University School of Medicine, Boston, Massachusetts, United States of America; New York University School of Medicine, United States of America

## Abstract

**Background:**

Regulatory T cells (Tregs) are essential in the control of tolerance. Evidence implicates Tregs in human autoimmune conditions. Here we investigated their role in systemic sclerosis (SSc).

**Methods/Principal Findings:**

Patients were subdivided as having limited cutaneous SSc (lcSSc, n = 20) or diffuse cutaneous SSc (dcSSc, n = 48). Further subdivision was made between early dcSSc (n = 24) and late dcSSc (n = 24) based upon the duration of disease. 26 controls were studied for comparison. CD3+ cells were isolated using FACS and subsequently studied for the expression of CD4, CD8, CD25, FoxP3, CD127, CD62L, GITR, CD69 using flow cytometry. T cell suppression assays were performed using sorted CD4CD25^high^CD127^-^ and CD4CD25^low^CD127^high^ and CD3^+^ cells. Suppressive function was correlated with CD69 surface expression and TGFβ secretion/expression. The frequency of CD4^+^CD25^+^ and CD25^high^FoxP3^high^CD127^neg^ T cells was highly increased in all SSc subgroups. Although the expression of CD25 and GITR was comparable between groups, expression of CD62L and CD69 was dramatically lower in SSc patients, which correlated with a diminished suppressive function. Co-incubation of Tregs from healthy donors with plasma from SSc patients fully abrogated suppressive activity. Activation of Tregs from healthy donors or SSc patients with PHA significantly up regulated CD69 expression that could be inhibited by SSc plasma.

**Conclusions/Significance:**

These results indicate that soluble factors in SSc plasma inhibit Treg function specifically that is associated with altered Treg CD69 and TGFβ expression. These data suggest that a defective Treg function may underlie the immune dysfunction in systemic sclerosis.

## Introduction

Over the past decade, there have been tremendous advances in our understanding of the basic processes that control immune tolerance. It is now generally accepted that auto-reactive T cells are present in healthy individuals, but that there mere presence does not necessitate the development of autoimmune disease. The identification of CD4+CD25+ regulatory T cells (Tregs) as a crucial component of self-tolerance has opened a major area of investigation and numerous studies have demonstrated the potent influence of Tregs in suppressing autoimmune disease, transplantation and graft-versus-host disease [1], [2], [3], [4], [5], [6], [7]. Studies in rodents have provided the first evidence for the existence of a naturally occurring population of CD4+CD25+ professional regulatory/suppressor T cells, which upon in vitro TCR-mediated stimulation, suppress proliferation of effector T cells [3], [8]. In the periphery of young mice not prone to autoimmune disease, Tregs constitute a stable 10% of CD4+ T cells. In contrast, mice genetically prone to autoimmune disease such as diabetes have markedly diminished circulating Tregs [9], [10].

Tregs have unique and robust immunosuppressive activity. The cells require specific TCR-mediated activation to develop regulatory capacity, but their effector function appears to be nonspecific, regulating local inflammatory responses through a combination of cell-cell contact and suppressive cytokine production [11], [12]. In addition to naturally occurring Tregs, several therapeutic interventions promote Treg development and function [13]. These so-called “adaptive” Treg populations share many features attributed to natural occurring Tregs, but can differ in critical cell surface markers [14].

In humans, the important role of Tregs in various autoimmune diseases has been underscored by numerous seminal studies. For instance, Tregs derived from patients with rheumatoid arthritis (RA) are defective in their ability to suppress cytokine production and to convey a suppressive phenotype to CD4+ effector T cells, which was at least partly restored upon treatment of TNFα neutralizing therapies [15]. Moreover, the interaction of Tregs with activated monocytes from patients with RA even led to a diminished suppressive activity possibly underlying their diminished capacity *in vivo* [16]. Likewise, it was demonstrated by several groups that the number and suppressive capacity of Tregs is altered in patients with systemic lupus erythematosus [17], [18], [19].

Systemic sclerosis (SSc) is a complex autoimmune disease characterized by an excessive deposition of matrix molecules, leading to fibrosis of multiple organs including the skin, lungs, heart and gastrointestinal tract, and often leading to severe morbidity and premature death. Although the role of immune dysfunction in the pathogenesis of SSc is currently not well understood, alterations in cellular immunity are typified by aberrant T cell biology both in the skin as well as circulation of SSc patients. For example, CD4+ T cells are increased in the circulation of SSc patients [20], [21], whereas NKT cells and γ/δ T cells are decreased [22]. In addition, lesional skin from SSc patients displays various features consistent with T cell activation [21], [23], [24]. Finally, circulating T cells from SSc patients show altered secretion of various inflammatory mediators compared to T cells from healthy controls [25], [26].

T cell priming by professional antigen presenting cells is tuned by an orchestra of inflammatory mediators, of which TGFβ, IL-23, IL-6, IL-22 and IL-1α are considered the most influential. For instance, in the absence of other pro-inflammatory mediators, TGFβ production by dendritic cells induces FoxP3, a Treg marker [27], [28]. In contrast, TGFβ in combination with IL-1α, IL-6 or IL-23 drives the expression of RORγT, a proliferation factor specific for the recently identified Th17 subset [29], [30], [31], [32]. Intriguingly, IL-23, IL-1α and IL-17 have been found increased in the circulation of SSc patients compared to healthy controls [33], [34], [35], [36]. Although TGFβ is not increased in SSc plasma, multiple studies have strongly implicated this cytokine as a major stimulus of fibrosis in involved organs. Together, these observations suggest that altered Treg function might play a key role in SSc pathogenesis. To address this issue, we set out to investigate changes in the number and/or function of Tregs in the peripheral blood of patients with SSc, taking into account the different disease phenotypes. In this paper, we show that Tregs are more frequent in SSc patients but are defective in their capacity to suppress proliferation of CD4+ effector T cells. We go on to demonstrate that this diminished suppressive effect of Tregs in SSc is associated with markedly lower expression of the activation marker CD69. Finally, we show that the diminished suppressive capacity and absent upregulation of CD69 upon activation is dependent upon soluble factors present in the plasma of SSc patients. Together these data suggest that diminished T regulatory capacity is present in SSc and that the regulatory deficiency is due to circulating factors rather than an inherent defect of Tregs.

## Methods

### Ethical review board statement

All samples were obtained with written informed consent after approval of the Institutional Review Board at the Boston University School of Medicine, Lund Univeristy medical Hospital and the Radboud University Nijmegen Medical Center.

#### Study population

Sixty-eight patients presenting to the Arthritis Center, Boston Medical Center were included in the study. This study was approved by the Boston University Medical Center Institutional Review Board. All of the patients met the American College of Rheumatology preliminary criteria for the classification of SSc [37]. Patients were subdivided as having limited cutaneous SSc (lcSSc, n = 20) or diffuse cutaneous SSc (dcSSc, n = 48) on the basis of the extent of their skin involvement [38]. A further subdivision was made between early dcSSc (n = 24) and late dcSSc (n = 24) based upon the duration of disease, defining early dcSSc as patients having a disease duration <2 years and late dcSSc as patients having a disease duration longer than 3 years. As a comparator group 26 healthy controls were studied. Patients were allowed to use low-dose prednisolone (<10 mg daily) at inclusion of the study. Patients receiving higher doses were excluded.

#### Monoclonal antibodies

For immunostaining and analysis by fluorescence-activated cell sorting (FACS), we used phycoerythrin (PE), allophycocyanin (APC) and fluorescein isothiocynate (FITC) conjugated mouse monoclonal antibodies (mAb) against human CD4, CD8, CD25, CD69, GITR (Miltenyi Biotec Inc., CA, USA), CD127 (eBioscience, CA, USA), CD62 (BD Bioscience, NJ, USA). Intracellular staining of CD4+CD25+ cells for FoxP3 was performed using the intracellular fixation and staining procedures according to the manufacturer's protocols. Corresponding mouse/rat isotype controls were included in the analyses.

#### Isolation of PBMCs, CD3^+^ cells and flowcytometry

PBMCs were isolated from heparinized venous blood by using density-gradient centrifugation over Ficoll-Paque (Amersham Bioscience). Next, CD3+ cells were isolated from PBMCs using CD3 microbeads according to manufacturer's protocol (Miltenyi Biotec). To this aim, 10 x 10^4^ CD3+ cells were re-suspended in 100 µl buffer (PBS + 1% BSA) on ice. After isolation, cells were directly transferred into RPMI 1640 media supplemented with 2nM L-glutamine, 100 U/µL/ml penicillin/streptomycin (Life technologies), and 10% FBS (BioWhitacker) in 96-well U-bottom plates (Nunc). For flowcytometric analysis, CD3+ were kept on ice and washed extensively with citrated PBS containing 1% FCS. Than, after using the protocol for fixation, intra-cellular staining was achieved using 10 µl of FITC, APC or PE- conjugated antibody that was added and incubated on ice for 20 min. 300 µl FACS buffer was than added and T cells were pelleted, resuspended in 200 µl buffer, and stained for the intracellular marker FoxP3/TGFβ were appropriate as conducted by the recommended procedure obtained from the manufacturer (Miltenyi Biotec Inc., CA, USA). Thereafter, cells were washed in buffer, fixed with 2% formaldehyde, washed again in buffer and stored at 4°C. The cells were analyzed using a LSRII FACScan flow cytometer (BD Biosciences) and data were processed using FlowJo software. In all experiments, the purity of CD3+, CD25^high^CD127^low^ and CD25^low^CD127^high^ cells was >97%.

#### Sorting of CD25^high^ and CD127^low^ cells for T cell suppression assays

For the T cell suppression assay, CD25^high^CD127^low^ cells were immediately incubated with CD25-PE and CD127-FITC (eBioscience, CA, USA) antibodies for 20 minutes on ice after CD3 MACS bead isolation. Thereafter, were sorted based upon the expression of CD25 and CD127. CD25^high^CD127^low^ cells, CD25^low^CD127^high^ cells and unsorted CD3+ T cells were transferred into RPMI 1640 media supplemented with 2nM L-glutamine, 100 U/µL/ml penicillin/streptomycin (Life technologies), and 10% FCS (BioWhitacker) in 96-well U-bottom plates (Nunc) until further use (overnight incubation). To assess the suppressive capacity of Treg (CD25^high^CD127^low^) and non-Treg (CD25^low^CD127^high^) cells on unsorted CD3+ cells, unsorted T cells were brought to a concentration of 2.10^6^ cells/ml and subsequently stimulated with phytohaemaglutinin (Sigma-Aldrich Corp, MO, USA). Both Tregs and non-Tregs from healthy controls and SSc patients were added to autologous unsorted CD3^+^ cells at fixed ratios 1∶20 for 5 consecutive days. After 4 d of culture, [3H]Tdr was added for the remaining 24 hrs of cultures. The cells were harvested onto glass fiber filters and [^3^H]thymidine incorporation was assessed on a beta scintillation counter.

#### Assessment of T cell suppressive effect and CD69 inducing capacity of SSc plasma

The effect of SSc plasma on the suppressive capacity of healthy Treg was investigated by co-incubation with 10% or 25% plasma from edSSc patients during whole experiment. For these experiments plasma was taken from the SSc patients and healthy controls at the same time point as the T cell experiments were performed. The plasma was stored at -80°C until further use. Plasma from 4 different edSSc patients was used in various independent experiments. To assess the CD69 inducing capacity of SSc plasma CD3+ cells and CD25^high^CD127^-^ cells were used from healthy controls and SSc patients. For this aim, both cell populations were cultured in RPMI 1640 media supplemented with 2nM L-glutamine, 100 U/µL/ml penicillin/streptomycin (Life technologies), and 10% FBS (BioWhitacker) in a 96 wells plate for 12 hours. Subsequently, CD3+ cells and CD25^high^CD127^-^ cells were stimulated with either phytohaemaglutinin (PHA) only, PHA in combination with 10% plasma from an early dcSSc or plasma alone. After 12 hours of stimulation cells were analyzed on expression of CD69 by flowcytometry as previously described.

#### Measurement of soluble and intracellular TGFβ

Intracellular TGFβ expression in CD25^high^CD127^-^ cells was investigated using a monoclonal antibody for TGFβ (BD Bioscience, NJ, USA) and the intracellular staining protocol as used for the FoxP3 staining. After the staining protocol, cells were fixed with 2% formaldehyde, stored at 4°C and analyzed on a flow cytometer the next day. TGFβ was assayed using mink lung epithelial cells stably transfected with a plasminogen activator inhibitor-1 promoter/luciferase reporter plasmid (provided by D. Rifkin) as described previously by Abe et al [39].

#### Statistical analysis

Values are shown throughout the paper as mean±sem. Proportions of lymphocyte subpopulations were compared using the Student's t test for normally or not normally distributed populations where appropriate. Relationships between different values were examined using Pearson's correlation coefficient and Spearman's rank correlation tests. All statistical analyses were performed using Graphpad Prism (GraphPad Prism 4.0 by Graph Pad software Inc.)

## Results

### CD4^+^CD25^+^FoxP3^+^CD127^-^ cells are markedly increased in the circulation of SSc patients irrespective of disease phenotype

**Figure 1:**
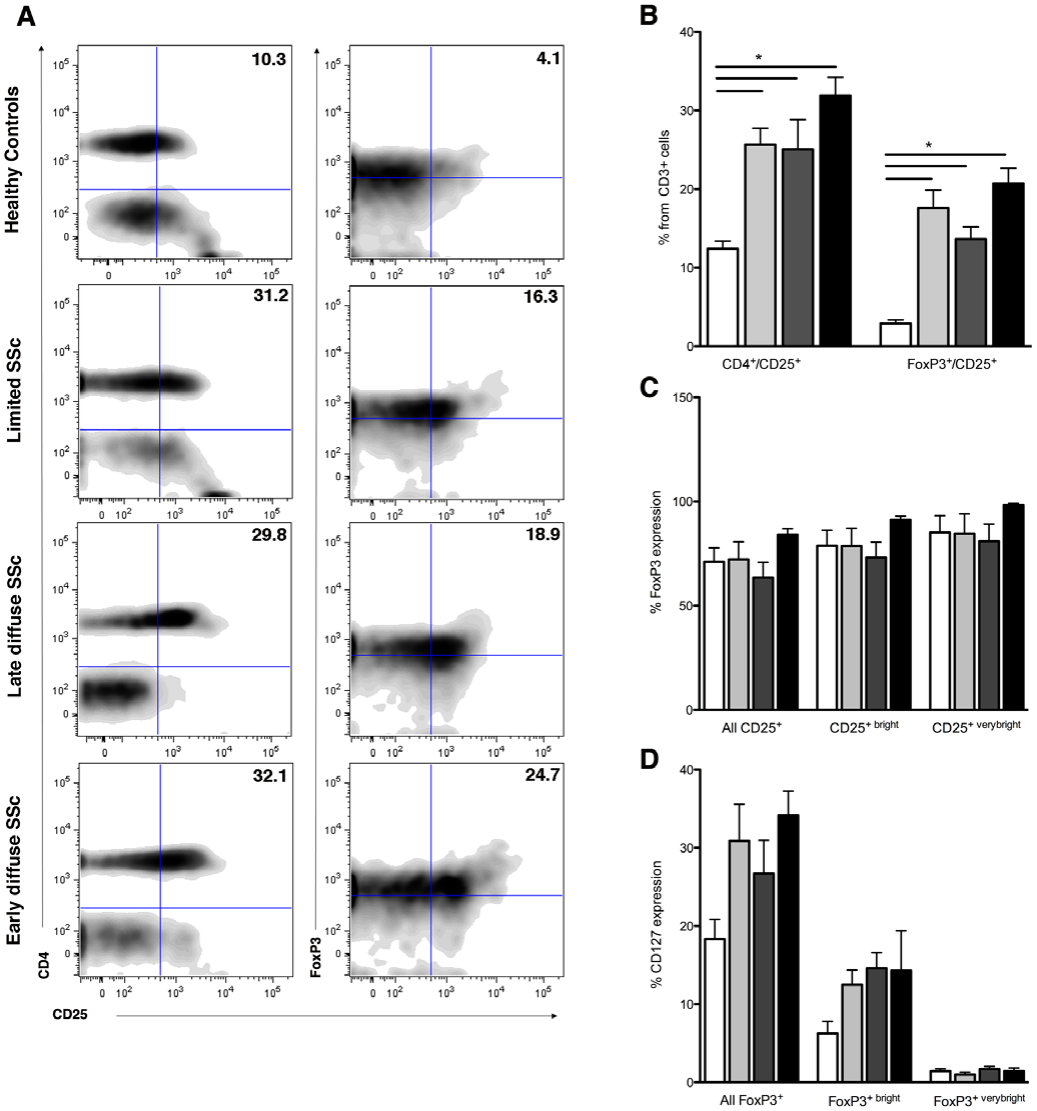
Increased presence of CD4^+^CD25^+^ and CD25^high^FoxP3^high^CD127^-^ cells in the circulation of patients with systemic sclerosis (SSc). Flow cytometry analysis of and CD4^+^CD25^+^ and CD25^high^FoxP3^high^CD127^-^ cells was performed in healthy controls (n = 26) and patients (n = 68) with different phenotypes of SSc. Peripheral blood mononuclear cells (PBMC's) were stained with anti-CD4, anti-CD25, anti-CD127 and anti-FoxP3, and analyzed by flow cytometry. (a) One representative individual from each group is shown. (b) Percentage of CD4+CD25+ and CD25+FoxP3+ cells are presented for each group, consisting of healthy controls (n = 26), lSSc (n = 20), ldSSc (n = 24) and edSSc (n = 24) patients. (c) Based upon CD25 expression, the top 10% (CD25^bright^) and top 2% (CD25^verybright^) were gated and FoxP3 expression analyzed as the percentage positive cells. (d) Based upon FoxP3 expression, the top 10% (FoxP3^bright^) and top 2% (FoxP3^verybright^) were gated and CD127 expression analyzed as the percentage positive cells. Data is presented as mean±sem.

Human peripheral blood contains a heterogeneous subset of CD4+CD25+ T cells that comprises T regulatory cells (Tregs) and a substantial number of activated effector T cells. To date, the expression of FoxP3 and CD127 remain the best and most specific markers of Tregs [40], [41]. Since we postulated that the number and/or phenotype of Tregs in SSc is altered compared to controls, but may also differ among different clinical SSc subtypes, we here studied the number and phenotype of Tregs from patients with limited cutaneous SSc (n = 20), late diffuse cutaneous SSc (n = 24) and early diffuse SSc (n = 24) in comparison with those from healthy controls (n = 26). The clinical characteristics of all patients included in this study are presented in **Table 1**. Despite similar absolute numbers of CD3+ cells, flowcytometric analysis with the markers CD4, CD25, FoxP3 and CD127, demonstrated that both CD4^+^CD25^+^ (12.4±1.0 *vs.* 27.5±2.8, *P*<0.0001) and CD25^+^FoxP3^+^CD127^-^ (2.9±0.5 *vs.* 17.3±1.9, *P*<0.0001) cells, (further designated as Tregs) are markedly increased in the circulation of SSc patients compared to controls (**Figure 1a, b** **)**. Further stratification to SSc disease phenotype revealed a significantly higher number of CD4^+^CD25^+^ (*P* = 0.01) and CD25^+^FoxP3^+^CD127^-^ (*P* = 0.01) in SSc patients with edSSc compared to ldSSc (**Figure 1b** **)**, but no other significant differences between SSc phenotypes were detected. Notably, two patients with ldSSc and two with edSSc received cyclophosphamide pulse therapy for their disease. Whereas both ldSSc responded clinically well only one patient with edSSc did. In these three patients the percentage CD25^+^FoxP3^+^CD127^-^ cells was much lower (6.0±2.1) compared with the other patients that were not treated. The edSSc patient that received cyclophosphamide pulse therapy but did not show a clinical response showed a frequency of 22.8% CD25^+^FoxP3^+^CD127^-^ cells. All the patients had received cyclophosphamide longer than 3 months ago. Further analysis focusing on CD25+^bright^ (top 10%) and CD25+^very bright^ (top 2%) cells revealed a similar expression of the markers FoxP3 and CD127 among all individuals, both on the levels of percentage positive cells (**Figure 1c, d** **)**, as well on the mean fluorescence intensity (MFI, data not shown). Taken together, these data suggest that SSc patients have a markedly increased frequency of T regulatory cells, which is not related to an altered expression of markers characterizing Treg phenotype.

**Table 1:** Patients clinical characteristics.

	Limited cutaneous SSc	Late diffuse cutaneous SSc	Early diffuse cutaneous SSc
Number	20	24	24
N females (%)	12 (92)	11 (79)	12 (80)
Age at onset	43.8±13.4	38.6±12.3	48.1±9.2
Disease duration	9.5±9.8	8.3±6.2	1.2±0.8
ANA positivity	100%	60%	93%
mRSS at inclusion	not assessed	17.6±8.6*	23.4±8.7*
Pulmonary hypertension	30%	13%	8%
Lung fibrosis	20%	52%	34%
Current Therapies			
MMF	0%	36%	27%
Cyclophosphamide	0%	14%	13%
Prednisolone	23%	29%	47%
Hydroxychloroquine	15%	7%	0%
Anti-IL-3	0%	0%	7%
Methotrexate	0%	0%	0%
Tacrolimus	7%	0%	0%

* P value 0.03.

### Aberrant expression of phenotypic markers CD62L and CD69 on CD25+FoxP3^bright^ and CD25+FoxP3+^verybright^ from SSc patients

**Figure 2:**
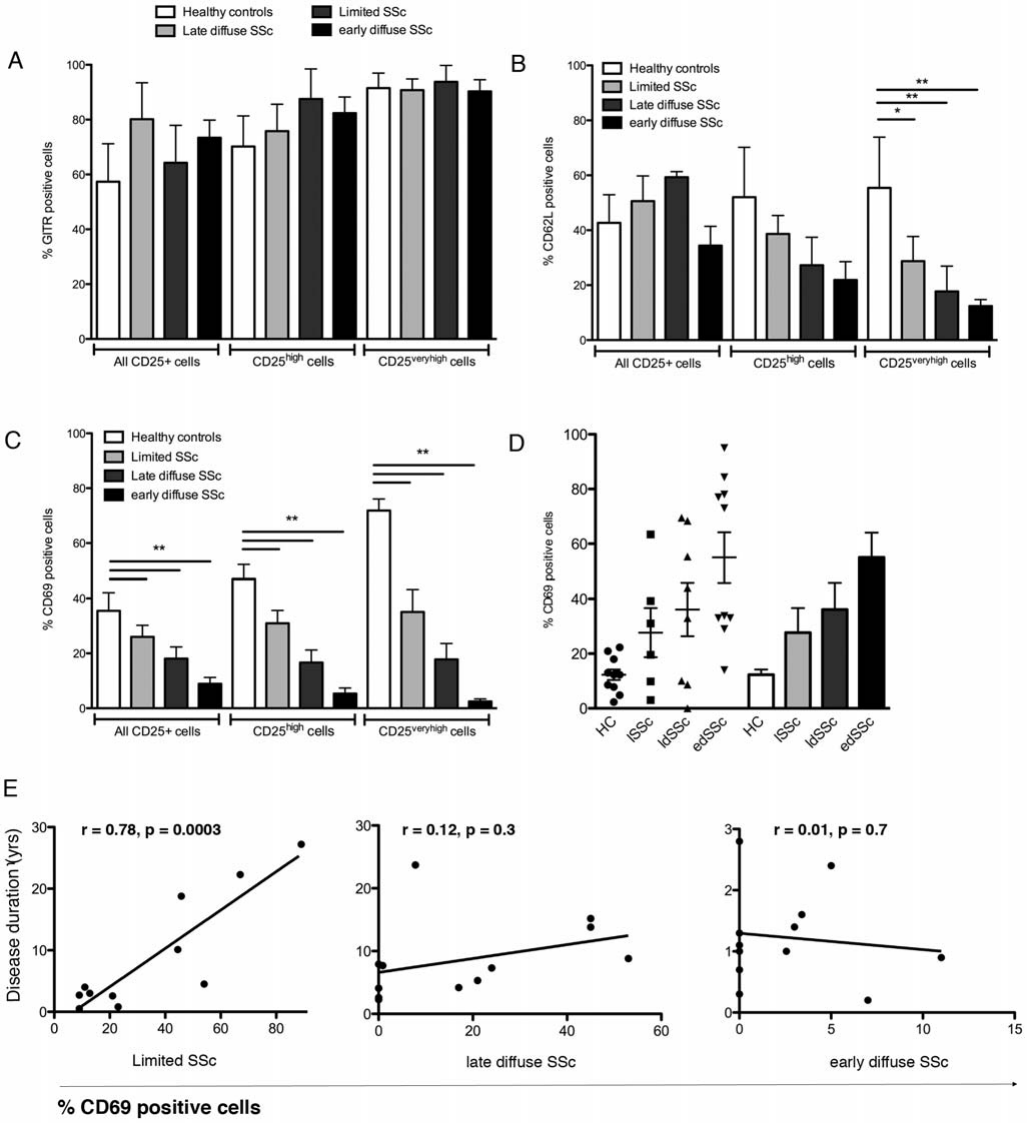
Phenotypical characterization of T regs reveals diminished expression of CD62L and CD69 in SSc patients. Panel (a) of this figure depicts the expression of the T cell activation marker GITR on CD25+, CD25^bright^ and CD25^verybright^ cells from healthy controls (white bars, n = 24) and SSc patients having limited cutaneous SSc (light gray bars, n = 18), late diffuse SSc (dark gray bars, n = 22) and early diffuse SSc (black bars, n = 22) patients. In panel (b) the expression of CD62L on Tregs is investigated. CD25+ and CD25^bright^ cells from SSc and healthy controls express similar levels of CD62L, whereas CD25^verybright^ from SSc patient subsets exhibit lower levels of CD62L compared to those from healthy controls. Panel (c) reflects the expression of CD69 on Tregs from healthy donors and SSc patients. CD25+, CD25^bright^ and CD25^verybright^ cells from SSc patients express significant lower levels of CD69 than those from healthy donors. CD69 expression on CD25^bright^ and CD25^verybright^ cells from edSSc patients was significantly lower then that from ldSSc patients, and ldSSc expressed CD69 significantly lower than those from lSSc. In panel (d) the expression on CD3+ cells is shown for all investigated groups. In contrast with that observed on Tregs from SSc patients, CD69 expression on CD4+ cells was significantly higher in all SSc patient groups. Panel (e) reflects the potential association between CD69 expression on Tregs and disease duration. CD69 expression on T regs from patients with lSSc correlated with disease duration, whereas this was not the case either with ldSSc nor edSSc. In all figures the white bars represent healthy controls, whereas lSSc, ldSSc and edSSc patients are represented by light gray, dark gray and black bars, respectively.

Although we observed a markedly increased frequency of CD25^+^FoxP3^+^CD127^-^ cells phenotypically representing Tregs in SSc, these patients continue to have active disease suggesting altered T cell suppressive activity. To address this, we next investigated the expression of markers potentially reflecting T cell activation including GITR, CD62L and CD69. Although the function of glucocorticoid-induced tumor necrosis factor receptor related protein (GITR) remains to be fully elucidated, it is generally accepted that GITR expression is increased upon TCR engagement, reflecting T cell activation [42]. As expected, GITR expression on CD25+Foxp3+, CD25+FoxP3+^bright^ and CD25+FoxP3+^verybright^ from healthy donors gradiently increased using flowcytometry **(** **Figure 2a** **)**. In addition, the expression of GITR on CD25+FoxP3+, CD25+FoxP3+^bright^ and CD25+FoxP3+^verybright^ was comparable between healthy controls and SSc patients and among the investigated SSc phenotypes. In contrast, the expression of CD62L and CD69 was markedly lower in SSc patients compared to healthy controls **(** **Figure 2b, c** **)**. CD62L is a L-selectin that is upregulated upon Treg activation and highly critical for Tregs to enter the lymph node and to carry out their local suppressive function [43], [44]. CD69 expression is pivotal for Treg function, potentially via upregulation of TGFβ production upon cross-linking [45], [46]. CD69 on CD25^high^ (37.0±5 *vs.* 17.6±5 *vs.* 5.3±2) and CD25^veryhigh^ (35.1±8 *vs.* 17.8±5.7 *vs.* 2.4±0.9) T cells significantly decreased in a step-like manner, comparing healthy controls to patients with lSSc, ldSSc and edSSc phenotypes. Intriguingly, and in line with that observed in other autoimmune diseases, the expression of CD69 on CD4^+^ effector T cells was significantly increased in all SSc patients compared to controls and followed an inverse correlation with the CD69 expression on CD25^high^ or FoxP3^high^ cells suggesting, that the regulation of CD69 expression is specifically altered on the Treg population in SSc [47], [48], [49] (**Figure 2d** **)**. In the search for potential SSc characteristics that might correlate with CD69 on Tregs in SSc, we found a significant association between the disease duration in lSSc patients whereas no association was present in patients either with ldSSc or edSSc **(** **Figure 2e** **)**.

### Diminished suppressive capacity of CD25^+^FoxP3^+^CD127^-^ regulatory T cells from SSc patients is correlated with CD69 expression and TGFβ levels

**Figure 3:**
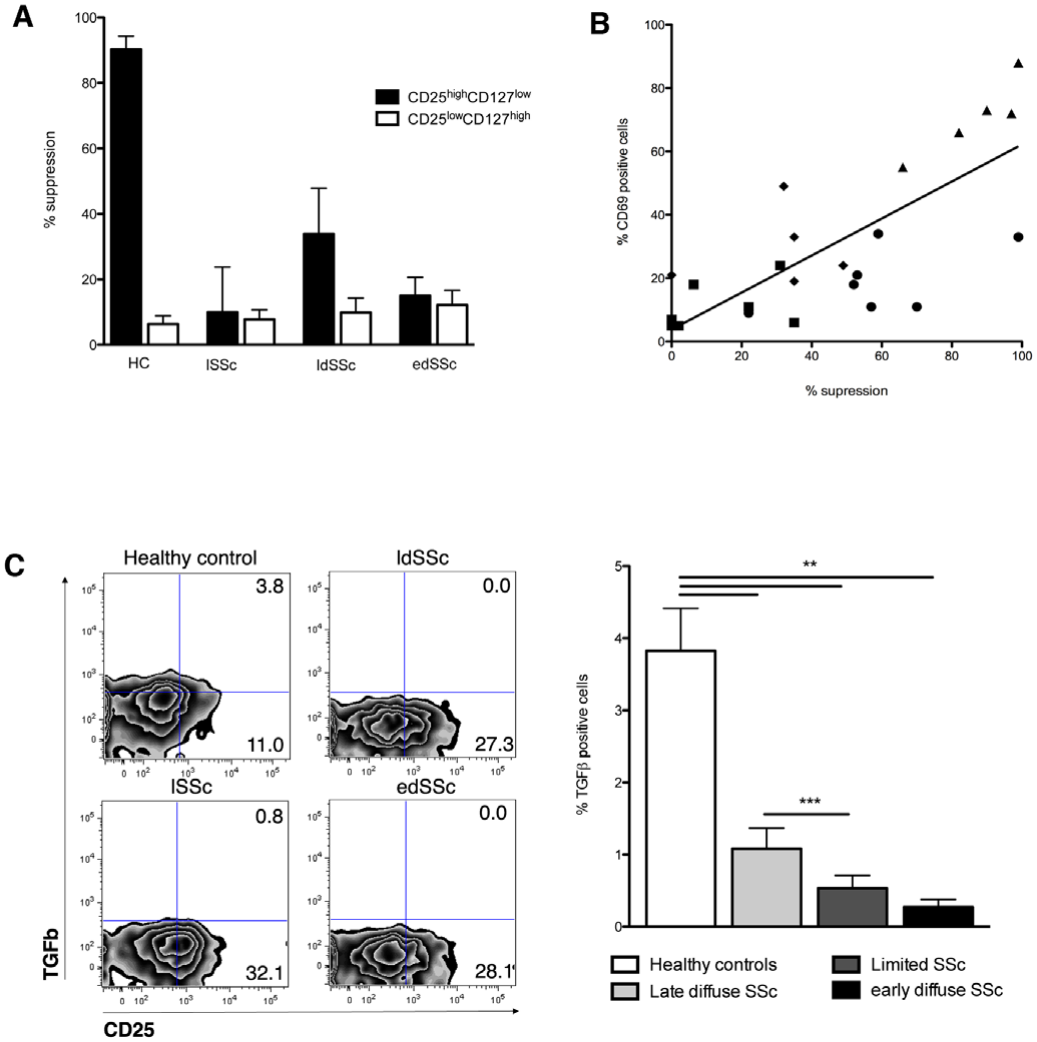
Impaired suppressive function by Tregs from SSc patients correlates with surface expression of CD69 and intracellular expression of TGFβ. Unsorted CD3+ (MACS bead isolated) were stimulated with PHA (5 µg/ml) and consecutively incubated with CD25^high^CD127^-^ or CD25^low^CD127^high^ cells for 5 days. Thereafter, CD3+ cells were incubated with 3^H^-thymidine for 24 more hours after which 3^H^-thymidine incorporation was measured. Panel (a) reflects the suppressive capacity of Tregs from healthy donors and SSc patients. Proliferation of CD3+ effector cells was effectively inhibited by T regulatory cells from healthy controls, whereas a clearly diminished suppressive activity was observed in the experiments with Tregs from SSc patients. Suppressive effect of Treg (CD25^high^CD127^-^) and non-Tregs (CD25^low^CD127^high^) is presented in black and white bars, respectively. Results are the mean and SEM of 6 separate experiments using cells from healthy donors (n = 9), lSSc (n = 7), ldSSc (n = 9) and edSSc (n = 7). Panel (b) represents the correlation of CD69 expression and Treg suppressive capacity in Tregs from the various groups under investigation. The percentage of CD69 positive regulatory T cells (CD25^high^CD127^-^) correlates well with the percentage of inhibition of CD3+ cells in healthy controls (triangles), lSSc (diamonds), ldSSc (circles) and edSSc (squares). Panel (c) reflects the expression of intracellular TGFβ in Tregs from healthy controls and SSc patients as measured using intracellular flow cytometry. CD25^high^CD127^-^ cells from all SSc patients express lower TGFβ levels compared to controls. Left panel reflects an representative individual from each group whereas the right panel displays the mean of each group comprising 6 individuals (per group) coming forth from 4 independent experiments.

Taken together, our observations imply that although SSc patients have a significantly increased number of CD25^+^FoxP3^+^CD127^-^ cells in the circulation, these cells phenotypically have markers suggesting impaired suppressive activity. To test the regulatory activity of these cells, we studied the capacity of CD25^high^CD127^low^ cells from healthy controls (n = 8), lSSc (n = 6), ldSSc (n = 9) and edSSc (n = 8) patients to suppress the proliferation of CD4^+^ effector cells. As expected, Tregs from healthy controls efficiently suppressed the proliferation of CD4+ effector cells by 87.3%±4.9, whereas non-regulatory T cells (CD25^low^CD127^high^) did not (8.5%±2.8). In contrast, Tregs obtained from SSc patients all had a markedly diminished suppressive capacity compared to those from healthy donors (**Figure 3a** **)** with T regs from lSSc, edSSc and ldSSc suppressing CD4+ effector cell proliferation by, respectively, 28.2%±6.0 (*P* = 0.0001), 56.0%±8.5 (*P* = 0.006) and 18.3%±5.2 (*P*<0.0001). Since CD69 expression by Tregs has been associated with the production of TGFβ [46], one of key molecules implicated in suppressor activity, we investigated the possible relationship between CD69 expression and the diminished suppressive effect observed in SSc. Interestingly, the suppressive capacity correlated significantly with CD69 expression in all groups **(** **Figure 3b** **)**.

We next investigated the expression levels of TGFβ in the Tregs from SSc patients compared to healthy controls and their CD45Ra+ cells. In line with the CD69, which was specifically lower on regulatory T cells in SSc, also TGFβ expression was significantly decreased by regulatory T cells obtained from SSc patients compared to those from healthy controls **(** **Figure 3c** **)**. TGFβ expression on Tregs from ldSSc and edSSc patients was significantly lower compared to that from patients with lSSc (*P* = 0.008), whereas no difference was observed between ldSSc and edSSc. Measurement of soluble TGFβ in the supernatant revealed no measurable TGFβ, suggesting that TGFβ confers its effect as membrane-bound (data not shown).

### A fraction smaller than 10kD in SSc plasma inhibits the suppressive capacity of regulatory T cells and abrogates the upregulation of CD69 specifically on regulatory T cells

**Figure 4:**
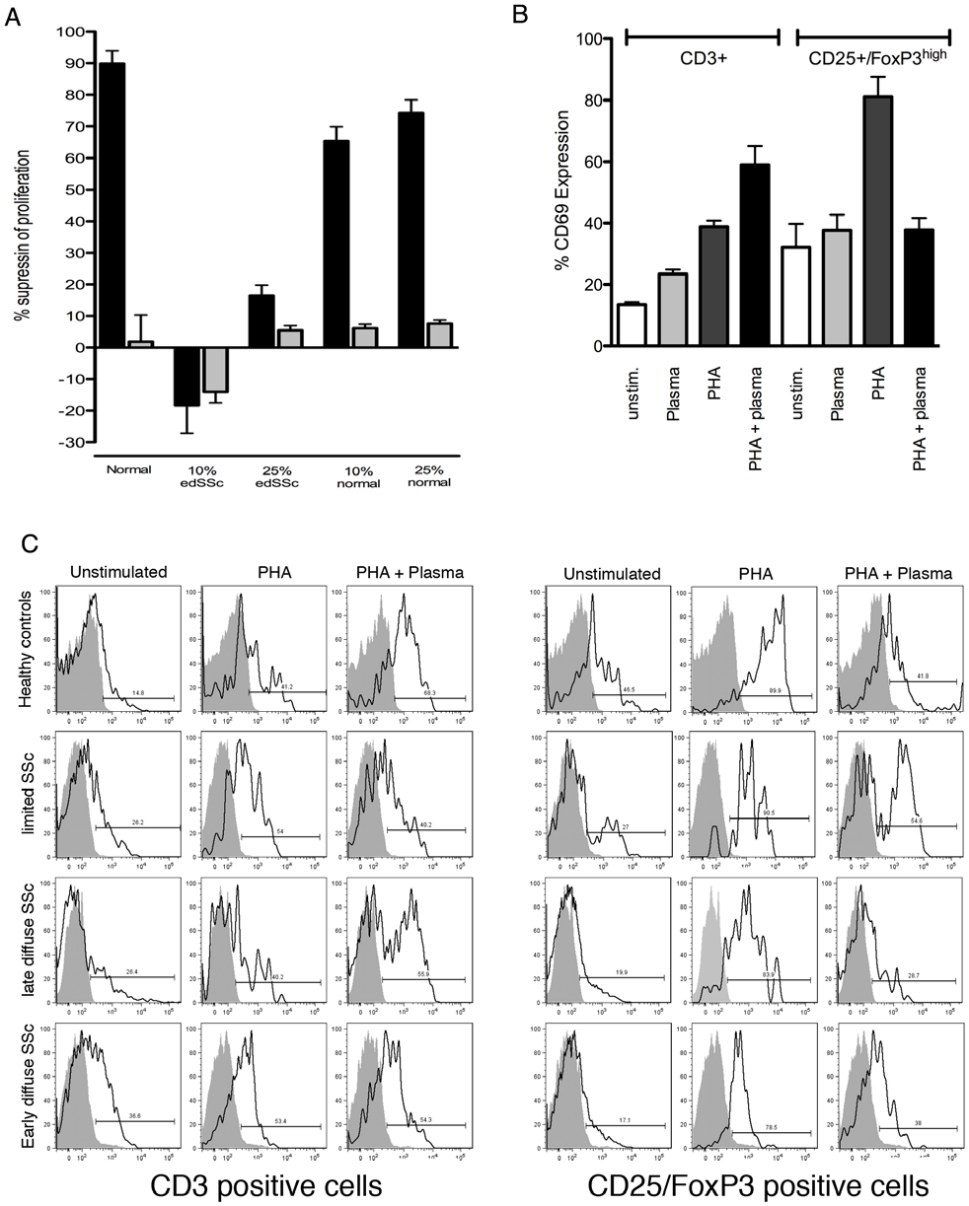
Plasma from SSc patients abrogates T cell suppression and up regulates CD69. (a) During the co-cultures of unsorted CD3+ cells with either Tregs (CD25^high^CD127^-^) or non-Tregs (CD25^low^CD127^high^) 10 or 25% plasma from an edSSc patient or healthy control was added to the culture. The graph represents data from 3 independent experiments using 3 healthy control cells, and plasma derived from two edSSc patients and two control individuals. (b) The effect of SSc plasma was evaluated by adding 10% to CD3+ cells for 24 hrs stimulated with PHA or unstimulated. As a control, CD69 expression was measured on CD3+ cells stimulated with PHA only. CD4 and CD25^high^/FoxP3^high^ cells were gated based on the expression of these markers using flow cytometry. (c) CD69 expression and induction upon PHA mediated stimulation of CD4+ and CD25^high^/FoxP3^high^ obtained from healthy donors, lSSc, ldSSc and edSSc patients was investigated using flow cytometry. One representative patient from each group is shown.

As inflammatory cytokines play an important role in the pathogenesis of SSc and regulatory T cell function, we next investigated whether the diminished suppressive effect of Tregs from SSc could be carried over by soluble factors in the circulation of SSc patients or alternatively could be due to an inherent defect in Tregs. Unexpectedly, the addition of 10% plasma from edSSc patients completely abrogated the suppressive capacity of Tregs on CD4+ effectors cells from healthy controls, an observation that was highly consistent throughout 5 experiments using plasma samples from 5 edSSc and 2 ldSSc patients **(** **Figure 4a** **)**. The addition of 25% plasma had a similar effect although somewhat less potent as 10% plasma, a phenomenon that was probably caused by the TGFβ present in patients plasma, that partly restored the suppressive capacity of Tregs. In contrast, the addition of plasma obtained from healthy controls did not have a significant effect on the suppressive capacity of Tregs.

Based on our observations that CD69 expression correlates with the diminished suppressive capacity in SSc, we hypothesized that the plasma of SSc patients had a direct effect on the regulation of CD69 expression. To test this, we stimulated freshly isolated CD3+ cells and CD25^high^CD127^low^ T cells from healthy controls with the potent T cell activator PHA. PHA markedly induced CD69 expression both on CD3+ and CD25^high^CD127^low^ T cells **(** **Figure 4b** **)**. However, plasma from edSSc patients also significantly increased CD69 expression on CD3+ cells (*P* = 0.0007) and had an additive effect in combination with PHA (*P* = 0.02). In contrast with the effect of plasma on CD3+ cells, the addition of edSSc plasma to CD25^high^CD127^low^ T cells did not increase CD69 expression. More intriguingly, the addition of SSc plasma to CD25^high^CD127^low^ T cells stimulated with PHA completely abrogated the effect of PHA. Since we observed a lower CD69 expression on regulatory T cells freshly isolated from SSc patients, we studied whether these cells still possess the ability to increase CD69 expression upon activation. Co-incubation of regulatory T cells either from patients with lSSc, ldSSc or edSSc led to an clear increase of CD69 expression that could be inhibited by plasma from edSSc patients **(** **Figure 4c** **)**. In all experiments presented here, plasma from healthy controls was taken into account but did sort any inhibitory effects as that observed from SSc patients.

## Discussion

SSc is an autoimmune disease that reflects several features suggesting dysregulated T cell activation [24], [50], [51]. The data presented here suggest that dysfunctional Tregs may play an important role in SSc. We show that although the number of Tregs is markedly increased in all clinical SSc phenotypes, these Tregs have a diminished capacity to control CD4 effector T cells. Further we show that their defective function correlates with lower expression of CD69 and TGFβ.

Tregs have not been previously characterized in patients with SSc; however, they are critical in maintaining self tolerance and preventing autoimmunity. In several other autoimmune disease Tregs have been implicated in pathogenesis. For example, lupus prone mice, depleted of CD4^+^CD25^+^ cells by thymectomy, have enhanced expansion of autoreactive T cells and accelerated autoantibody production [52]. Conversely, restoration of the CD4^+^CD25^+^ cell population from syngeneic normal mice effectively abrogates the development of autoimmune disease, as has treatment with in vitro expanded Tregs [52], [53], [54]. Similar evidence originates from experimental arthritis, diabetes and multiple sclerosis models, further highlighting the crucial role of the Tregs in controlling the delicate balance between tolerance and autoimmunity. More recently, several studies performed in patients with systemic lupus erythematosus (SLE) and rheumatoid arthritis (RA) revealed an aberrant frequency and/or function of Tregs thus indicating their crucial role in human diseases [18], [19], [55], [56], [57]. However, none of these studies reported the markedly increased frequency of CD4^+^CD25^+^ and CD25^+^/FoxP3^+^CD127^-^ cells found in our study. In contrast, although some inconsistencies exist, most of these studies found a decreased frequency of circulating Tregs. There appear to be some discrepancies in the literature based on the sole use of CD4 and CD25 as markers for Tregs. However, co-expression of CD4 and CD25 can be induced upon multiple inflammatory events and does not necessarily guarantee suppressive capacity. Therefore, the limited use of these markers could merely reflect activation and thus lead to a false assessment of elevated Treg numbers. More recently, it has been shown that the combination of FoxP3 and CD127 expression is highly specific for discriminating Tregs from activated T cells. FoxP3 expression correlated inversely with CD127 expression, and CD4+CD25^high^FoxP3^high^CD127^low^ cells were found to have the most potent suppressive activity [40], [58]. In the current study the combination of all these markers was used to characterize and isolate regulatory T cells, confirming our observations of a markedly increased frequency of circulating Tregs in SSc patients.

TGFβ is known to potently induce expression of the proliferation factor FoxP3, characterizing Tregs. As TGFβ is generally accepted as the key regulator of SSc pathogenesis, the increased frequency of Tregs in SSc was not surprising. TGFβ is crucial in the induction of FoxP3 expression and induction of suppressive activity by conversion of CD4+CD25- T cells [59]. Therefore, increased TGFβ found in SSc might drive the increased frequency of CD25^high^FoxP3^high^CD127^-^. Indeed, our observation of increased FoxP3, despite comparable levels of CD25 and GITR expression in SSc patients, suggests that Tregs from SSc patients are activated to some extent. The observation that CD62L, a marker that is highly expressed on naturally occurring (thymically-derived) regulatory T cells, is lower in SSc patients suggests that these Tregs originate through conversion of CD4^+^CD25^-^ T cells. These so-called “adaptive” Tregs share many features with naturally occurring Tregs, but can differ in critical cell surface biomarkers and functional attributes [14]. For instance, Tregs can mediate their suppressive effects through the production of IL-10 versus TGFβ [60], [61].

In contrast to CD25 and GITR expression, CD69 expression on Tregs was significantly lower in SSc patients and correlated closely with diminished suppressive activity. Further, upregulation of CD69 by T cell stimulation was completely abrogated by plasma from SSc patients, suggesting the presence of soluble factors in SSc plasma that inhibit CD69 and consequently, the suppressive capacity. Interestingly, the effect of plasma on CD69 expression was highly specific for Tregs, since CD69 regulation on other T cells was not affected. SSc patients show many features suggesting that autoimmune and inflammatory factors may stimulate profibrotic organ damage. For instance, accumulating evidence implicates inflammatory mediators in the Th17 pathway, such as IL-6, IL-1α, IL-23 and IL-17 itself, but also those in the Th2 (IL-10, IL-4), Th1 (IFNγ) and other inflammatory pathways, such as IFN type I and TNFα, in this condition (unpublished results [33], [34], [35], [36]). It is therefore tempting to speculate that several mediators could inhibit Treg CD69 expression in SSc patients. In this light, the observation that the three patients who had a clinical response to treatment had a Treg frequency, CD69 expression and suppressive capacity that was almost comparable to that observed in healthy controls is intriguing. Whether these observations are related to lower levels of inflammatory mediators in patients with a therapeutic response will require further investigation.

The potential of Tregs to modulate immune responses has led to considerable interest in their use for clinical intervention in autoimmune diseases. Two broad therapeutic applications have been considered: first, to expand the regulatory T cell compartment ex vivo with the goal of re-infusion and second, to manipulate the immune system in vivo resulting in an increase of Tregs. The latter approach has been shown to be highly applicable by seminal studies by Ehrenstein et al. in which a monoclonal antibody against TNFα led to a re-occurrence of CD4^+^CD25^+^CD62L^-^ T cells with high suppressive activity [15], [62]. Of interest for the current study the suppressive effects of Tregs in these latter studies were found to be contact dependent since the neutralization of TGFβ and IL-10 did not block the effect. This is consistent with our observation that intracellular expression of TGFβ on Tregs corresponded well with their suppressive capacity, whereas no TGFβ was found in the culture supernatants. In our studies we demonstrate that a soluble factors in the plasma of SSc patients is responsible for the dramatic effects observed on suppressive activity, CD69 and TGFβ expression. In addition, we did not find evidence for an inherent defect in lower Treg CD69 expression in SSc patients, since activation of these cells led to increased expression.

The factors driving TGFβ production are not well resolved. The role of CD69 in the production of TGFβ by T cells was shown in several studies. For instance, it was demonstrated that CD69-/- mice display greatly prolonged tumor survival that was related to a decreased production of TGFβ. CD69 engagement induced TGFβ production by NK and T cells [63]. With respect to autoimmunity, CD69-/- mice showed a higher incidence and severity of collagen-induced arthritis, which again were correlated with reduced levels of TGFβ [46]. The observation that CD69 surface expression closely mirrors intra-cellular TGFβ expression both on CD45Ra as on CD25^high^FoxP3^high^CD127^-^ cells is in line with the notion that CD69 is implicated in TGFβ production by T regs.

Altogether, our observations provide a rationale for therapeutic intervention to restore suppressive activity by T regs in SSc. More careful studies designed to identify the nature of factors that moderate the effects in the circulation are warranted.

## References

[1] Chatenoud L, Salomon B, Bluestone JA (2001). Suppressor T cells–they're back and critical for regulation of autoimmunity!. Immunol Rev.

[2] Curotto de Lafaille MA, Lafaille JJ (2002). CD4(+) regulatory T cells in autoimmunity and allergy.. Curr Opin Immunol.

[3] Sakaguchi S, Takahashi T, Yamazaki S, Kuniyasu Y, Itoh M (2001). Immunologic self tolerance maintained by T-cell-mediated control of self-reactive T cells: implications for autoimmunity and tumor immunity.. Microbes Infect.

[4] Singh B, Read S, Asseman C, Malmstrom V, Mottet C (2001). Control of intestinal inflammation by regulatory T cells.. Immunol Rev.

[5] Takahashi T, Tagami T, Yamazaki S, Uede T, Shimizu J (2000). Immunologic self-tolerance maintained by CD25(+)CD4(+) regulatory T cells constitutively expressing cytotoxic T lymphocyte-associated antigen 4.. J Exp Med.

[6] Tang Q, Boden EK, Henriksen KJ, Bour-Jordan H, Bi M (2004). Distinct roles of CTLA-4 and TGF-beta in CD4+CD25+ regulatory T cell function.. Eur J Immunol.

[7] Wood KJ, Sakaguchi S (2003). Regulatory T cells in transplantation tolerance.. Nat Rev Immunol.

[8] Bach JF (2003). Regulatory T cells under scrutiny.. Nat Rev Immunol.

[9] Salomon B, Lenschow DJ, Rhee L, Ashourian N, Singh B (2000). B7/CD28 costimulation is essential for the homeostasis of the CD4+CD25+ immunoregulatory T cells that control autoimmune diabetes.. Immunity.

[10] Wu AJ, Hua H, Munson SH, McDevitt HO (2002). Tumor necrosis factor-alpha regulation of CD4+CD25+ T cell levels in NOD mice.. Proc Natl Acad Sci U S A.

[11] Shevach EM (2008). Special regulatory T cell review: How I became a T suppressor/regulatory cell maven.. Immunology.

[12] von Boehmer H (2005). Mechanisms of suppression by suppressor T cells.. Nat Immunol.

[13] Belghith M, Bluestone JA, Barriot S, Megret J, Bach JF (2003). TGF-beta-dependent mechanisms mediate restoration of self-tolerance induced by antibodies to CD3 in overt autoimmune diabetes.. Nat Med.

[14] Bluestone JA, Abbas AK (2003). Natural versus adaptive regulatory T cells.. Nat Rev Immunol.

[15] Ehrenstein MR, Evans JG, Singh A, Moore S, Warnes G (2004). Compromised function of regulatory T cells in rheumatoid arthritis and reversal by anti-TNFalpha therapy.. J Exp Med.

[16] van Amelsfort JM, van Roon JA, Noordegraaf M, Jacobs KM, Bijlsma JW (2007). Proinflammatory mediator-induced reversal of CD4+,CD25+ regulatory T cell-mediated suppression in rheumatoid arthritis.. Arthritis Rheum.

[17] Jaeckel E, Mpofu N, Saal N, Manns MP (2008). Role of regulatory T cells for the treatment of type 1 diabetes mellitus.. Horm Metab Res.

[18] Yan B, Ye S, Chen G, Kuang M, Shen N (2008). Dysfunctional CD4+,CD25+ regulatory T cells in untreated active systemic lupus erythematosus secondary to interferon-alpha-producing antigen-presenting cells.. Arthritis Rheum.

[19] Bonelli M, von Dalwigk K, Savitskaya A, Smolen JS, Scheinecker C (2008). Foxp3 expression in CD4+ T cells of patients with systemic lupus erythematosus: a comparative phenotypic analysis.. Ann Rheum Dis.

[20] Gustafsson R, Totterman TH, Klareskog L, Hallgren R (1990). Increase in activated T cells and reduction in suppressor inducer T cells in systemic sclerosis.. Ann Rheum Dis.

[21] Hussein MR, Hassan HI, Hofny ER, Elkholy M, Fatehy NA (2005). Alterations of mononuclear inflammatory cells, CD4/CD8+ T cells, interleukin 1beta, and tumour necrosis factor alpha in the bronchoalveolar lavage fluid, peripheral blood, and skin of patients with systemic sclerosis.. J Clin Pathol.

[22] Riccieri V, Parisi G, Spadaro A, Scrivo R, Barone F (2005). Reduced circulating natural killer T cells and gamma/delta T cells in patients with systemic sclerosis.. J Rheumatol.

[23] Kalogerou A, Gelou E, Mountantonakis S, Settas L, Zafiriou E (2005). Early T cell activation in the skin from patients with systemic sclerosis.. Ann Rheum Dis.

[24] Parel Y, Aurrand-Lions M, Scheja A, Dayer JM, Roosnek E (2007). Presence of CD4+CD8+ double-positive T cells with very high interleukin-4 production potential in lesional skin of patients with systemic sclerosis.. Arthritis Rheum.

[25] Del Galdo F, Jimenez SA (2007). T cells expressing allograft inflammatory factor 1 display increased chemotaxis and induce a profibrotic phenotype in normal fibroblasts in vitro.. Arthritis Rheum.

[26] Fujii H, Hasegawa M, Takehara K, Mukaida N, Sato S (2002). Abnormal expression of intracellular cytokines and chemokine receptors in peripheral blood T lymphocytes from patients with systemic sclerosis.. Clin Exp Immunol.

[27] Liu Y, Zhang P, Li J, Kulkarni AB, Perruche S (2008). A critical function for TGF-beta signaling in the development of natural CD4+CD25+Foxp3+ regulatory T cells.. Nat Immunol.

[28] Luo X, Tarbell KV, Yang H, Pothoven K, Bailey SL (2007). Dendritic cells with TGF-beta1 differentiate naive CD4+CD25- T cells into islet-protective Foxp3+ regulatory T cells.. Proc Natl Acad Sci U S A.

[29] Annunziato F, Cosmi L, Santarlasci V, Maggi L, Liotta F (2007). Phenotypic and functional features of human Th17 cells.. J Exp Med.

[30] Manel N, Unutmaz D, Littman DR (2008). The differentiation of human T(H)-17 cells requires transforming growth factor-beta and induction of the nuclear receptor RORgammat.. Nat Immunol.

[31] Volpe E, Servant N, Zollinger R, Bogiatzi SI, Hupe P (2008). A critical function for transforming growth factor-beta, interleukin 23 and proinflammatory cytokines in driving and modulating human T(H)-17 responses.. Nat Immunol.

[32] Wilson NJ, Boniface K, Chan JR, McKenzie BS, Blumenschein WM (2007). Development, cytokine profile and function of human interleukin 17-producing helper T cells.. Nat Immunol.

[33] Duan H, Fleming J, Pritchard DK, Amon LM, Xue J (2008). Combined analysis of monocyte and lymphocyte messenger RNA expression with serum protein profiles in patients with scleroderma.. Arthritis Rheum.

[34] Komura K, Fujimoto M, Hasegawa M, Ogawa F, Hara T (2007). Increased Serum Interleukin 23 in Patients with Systemic Sclerosis.. J Rheumatol.

[35] Kurasawa K, Hirose K, Sano H, Endo H, Shinkai H (2000). Increased interleukin-17 production in patients with systemic sclerosis.. Arthritis Rheum.

[36] Murata M, Fujimoto M, Matsushita T, Hamaguchi Y, Hasegawa M (2008). Clinical association of serum interleukin-17 levels in systemic sclerosis: Is systemic sclerosis a Th17 disease?. J Dermatol Sci.

[37] (1980). Preliminary criteria for the classification of systemic sclerosis (scleroderma). Subcommittee for scleroderma criteria of the American Rheumatism Association Diagnostic and Therapeutic Criteria Committee.. Arthritis Rheum.

[38] LeRoy EC, Medsger TA (2001). Criteria for the classification of early systemic sclerosis.. J Rheumatol.

[39] Abe M, Harpel JG, Metz CN, Nunes I, Loskutoff DJ (1994). An assay for transforming growth factor-beta using cells transfected with a plasminogen activator inhibitor-1 promoter-luciferase construct.. Anal Biochem.

[40] Liu W, Putnam AL, Xu-Yu Z, Szot GL, Lee MR (2006). CD127 expression inversely correlates with FoxP3 and suppressive function of human CD4+ T reg cells.. J Exp Med.

[41] Ziegler SF (2006). FOXP3: of mice and men.. Annu Rev Immunol.

[42] Shevach EM, Stephens GL (2006). The GITR-GITRL interaction: co-stimulation or contrasuppression of regulatory activity?. Nat Rev Immunol.

[43] Huehn J, Siegmund K, Lehmann JC, Siewert C, Haubold U (2004). Developmental stage, phenotype, and migration distinguish naive- and effector/memory-like CD4+ regulatory T cells.. J Exp Med.

[44] Siegmund K, Feuerer M, Siewert C, Ghani S, Haubold U (2005). Migration matters: regulatory T-cell compartmentalization determines suppressive activity in vivo.. Blood.

[45] Ishikawa S, Akakura S, Abe M, Terashima K, Chijiiwa K (1998). A subset of CD4+ T cells expressing early activation antigen CD69 in murine lupus: possible abnormal regulatory role for cytokine imbalance.. J Immunol.

[46] Sancho D, Gomez M, Viedma F, Esplugues E, Gordon-Alonso M (2003). CD69 downregulates autoimmune reactivity through active transforming growth factor-beta production in collagen-induced arthritis.. J Clin Invest.

[47] Afeltra A, Galeazzi M, Ferri GM, Amoroso A, De Pita O (1993). Expression of CD69 antigen on synovial fluid T cells in patients with rheumatoid arthritis and other chronic synovitis.. Ann Rheum Dis.

[48] Fernandez-Gutierrez B, Hernandez-Garcia C, Banares AA, Jover JA (1995). Characterization and regulation of CD69 expression on rheumatoid arthritis synovial fluid T cells.. J Rheumatol.

[49] Portales-Perez D, Gonzalez-Amaro R, Abud-Mendoza C, Sanchez-Armass S (1997). Abnormalities in CD69 expression, cytosolic pH and Ca2+ during activation of lymphocytes from patients with systemic lupus erythematosus.. Lupus.

[50] Boin F, Wigley FM, Schneck JP, Oelke M, Rosen A (2005). Evaluation of topoisomerase-1-specific CD8+ T-cell response in systemic sclerosis.. Ann N Y Acad Sci.

[51] Scherer HU, Burmester GR, Riemekasten G (2006). Targeting activated T cells: successful use of anti-CD25 monoclonal antibody basiliximab in a patient with systemic sclerosis.. Ann Rheum Dis.

[52] Bagavant H, Tung KS (2005). Failure of CD25+ T cells from lupus-prone mice to suppress lupus glomerulonephritis and sialoadenitis.. J Immunol.

[53] Scalapino KJ, Tang Q, Bluestone JA, Bonyhadi ML, Daikh DI (2006). Suppression of disease in New Zealand Black/New Zealand White lupus-prone mice by adoptive transfer of ex vivo expanded regulatory T cells.. J Immunol.

[54] Zheng SG, Wang JH, Koss MN, Quismorio F, Gray JD (2004). CD4+ and CD8+ regulatory T cells generated ex vivo with IL-2 and TGF-beta suppress a stimulatory graft-versus-host disease with a lupus-like syndrome.. J Immunol.

[55] Chowdary Venigalla RK, Tretter T, Krienke S, Max R, Eckstein V (2008). Reduced CD4+,CD25- T cell sensitivity to the suppressive function of CD4+,CD25(high),CD127(-/low) regulatory T cells in patients with active systemic lupus erythematosus.. Arthritis Rheum.

[56] Lee HY, Hong YK, Yun HJ, Kim YM, Kim JR (2008). Altered frequency and migration capacity of CD4+CD25+ regulatory T cells in systemic lupus erythematosus.. Rheumatology (Oxford).

[57] Nadkarni S, Mauri C, Ehrenstein MR (2007). Anti-TNF-alpha therapy induces a distinct regulatory T cell population in patients with rheumatoid arthritis via TGF-beta.. J Exp Med.

[58] Seddiki N, Santner-Nanan B, Martinson J, Zaunders J, Sasson S (2006). Expression of interleukin (IL)-2 and IL-7 receptors discriminates between human regulatory and activated T cells.. J Exp Med.

[59] Zheng SG, Gray JD, Ohtsuka K, Yamagiwa S, Horwitz DA (2002). Generation ex vivo of TGF-beta-producing regulatory T cells from CD4+CD25- precursors.. J Immunol.

[60] Asseman C, Mauze S, Leach MW, Coffman RL, Powrie F (1999). An essential role for interleukin 10 in the function of regulatory T cells that inhibit intestinal inflammation.. J Exp Med.

[61] Fukaura H, Kent SC, Pietrusewicz MJ, Khoury SJ, Weiner HL (1996). Induction of circulating myelin basic protein and proteolipid protein-specific transforming growth factor-beta1-secreting Th3 T cells by oral administration of myelin in multiple sclerosis patients.. J Clin Invest.

[62] Nadkarni S, Jung P (2004). Dressed neurons: modeling neural-glial interactions.. Phys Biol.

[63] Esplugues E, Sancho D, Vega-Ramos J, Martinez C, Syrbe U (2003). Enhanced antitumor immunity in mice deficient in CD69.. J Exp Med.

